# Bibliometric and visualized analysis of elite controllers based on CiteSpace: landscapes, hotspots, and frontiers

**DOI:** 10.3389/fcimb.2023.1147265

**Published:** 2023-04-12

**Authors:** Xingyue Yuan, Yu Lai

**Affiliations:** ^1^School of Clinical Medicine, Chengdu University of Traditional Chinese Medicine, Chengdu, China; ^2^School of Basic Medicine, Chengdu University of Traditional Chinese Medicine, Chengdu, China

**Keywords:** HIV, elite controllers, CiteSpace, bibliometric analysis, visualized analysis, research hotspots

## Abstract

**Background:**

A unique subset of people living with HIV, known as elite controllers, possess spontaneous and consistent control over viral replication and disease progression in the absence of antiviral intervention. In-depth research on elite controllers is conducive to designing better treatment strategies for HIV. However, comprehensive and illuminating bibliometric reports on elite controllers are rare.

**Methods:**

Articles on elite controllers were retrieved from the Web of Science Core Collection. A visualized analysis of this domain was conducted by CiteSpace software. Taking count, betweenness centrality, and burst value as criteria, we interpreted the visualization results and predicted future new directions and emerging trends.

**Results:**

By December 31, 2022, 843 articles related to elite controllers had been published. The largest contributors in terms of country, institution, and author were the United States (485), Univ Calif San Francisco (87), and Walker B.D. (65), respectively. Migueles S.A. (325) and *Journal of Virology* (770) were the most cocited author and journal, respectively. Additionally, by summarizing the results of our CiteSpace software analysis on references and keywords, we considered that the research hotspots and frontiers on elite controllers mainly focus on three aspects: deciphering the mechanisms of durable control, delineating the implications for the development of treatments for HIV infection, and highlighting the clinical risks faced by elite controllers and coping strategies.

**Conclusion:**

This study performed a bibliometric and visual analysis of elite controllers, identified the main characteristics and emerging trends, and provided insightful references for further development of this rapidly evolving and complex field.

## 1 Introduction

Although the first case of acquired immune deficiency syndrome (AIDS) was reported in the United States in 1981, the oldest documented human immunodeficiency virus (HIV) infection to date was discovered in a 1959 plasma sample from Belgian Congo (Zhu et al., 1998). This is a severe contagious virus that has spread at an alarming rate worldwide. It poses a severe threat to human health with high incidence of mortality if left untreated. The total number of people living with HIV reached approximately 37.7 million globally at the end of 2020, including 1.5 million new infections in 2020, and an estimated 680 000 deaths due to HIV and AIDS-related illnesses. (WHO, 2022).

Due to HIV-1’s capability to latently infect CD4^+^ T cells and form a long-lived and replication-competent latent viral reservoir that cannot be eliminated by antiretroviral therapy (ART), people living with HIV (PLHIV) often need to make a lifetime commitment to suppress plasma viral loads to undetectable levels by taking antiretroviral medication. Viral replication will quickly rebound once the drugs are interrupted (Chun et al., 1997). The rapid mutation and recombination of HIV, amongst other things, presents significant obstacles to designing a universal vaccine (Ng'uni et al., 2020). Bone marrow transplantation as a cure for HIV is not an ideal treatment for HIV, mainly owing to its high cost, substantial risks, and difficulty in finding matched donors with homozygous CCR5Δ32 gene mutations that protect against HIV acquisition. Therefore, we urgently need to discover new practicable clues for developing future treatment strategies toward a functional cure for HIV.

Among PLHIV, there is a distinctive subgroup termed elite controllers (ECs), who possess spontaneous and drug-free virologic control to maintain undetectable viral loads (plasma HIV RNA levels <50 copies/mL) for extended periods despite the existence of latent viral reservoirs. Meanwhile, they also tend to have more stable CD4^+^ cell count and a lower risk of developing AIDS-related illnesses (Okulicz et al., 2009). Less than 1% of PLHIV worldwide are born with this rare immunological ability to suppress HIV replication without antiviral medication (Okulicz and Lambotte, 2011). During the past 20 years, a number of studies have shown that some common characteristics exist among elite controllers, which may help us to obtain a better understanding of the immunological factors that may contribute to a functional cure for HIV (Autran et al., 2011; Tarancon-Diez et al., 2018). To date, two ECs, also referred to as the “San Francisco patient” reported in 2020 and the “Esperanza patient” reported in 2022, have persistently shown to have undetectable levels of genome-intact HIV-1 provirus in more than a billion peripheral blood mononuclear cells for years without the use of antiviral medications or stem cell transplantation, suggesting that an HIV sterilizing cure through natural immunity may be an extremely rare but feasible outcome (Jiang et al., 2020; Turk et al., 2022). Understanding the commonality of virological and immunological mechanisms by which the virus is naturally and persistently suppressed in elite controllers could help design HIV therapeutic strategies that aim at a functional cure in a broader population.

CiteSpace is a Java-based cocitation network visualization application, developed by Professor Chao-mei Chen in 2004, to detect and reveal research fronts and abrupt turning points in a knowledge domain (Chen, 2006). It has been used to conduct hundreds of bibliometric studies (Chen et al., 2014). By monitoring and processing the information from databases, CiteSpace can present the knowledge of a scientific subject in terms of scientific cooperation networks, citation networks, cocitation networks, and coauthorship social networks in the form of a scientific network (Chen, 2004). The findings of the CiteSpace analysis provide a systematic analysis of a rapidly evolving and complex field to enable analysts who lack domain expertise to identify and analyze emerging trends and transitional patterns (Chen et al., 2014).

As far as we know, no comprehensive and illuminating bibliometric reports on elite controllers exist. To understand the research status and new trends in the elite controller field and to provide specific ideas and references for the development of HIV treatment research in the future, we used CiteSpace 5.7.r5 software to draw mapping knowledge domains (MKD) and interpreted the hot-spot topics and frontier trends.

## 2 Methods

### 2.1 Data collection

We retrieved all relevant documents from the Web of Science Core Collection (WOSCC) to December 31, 2022, and the initial year of the time span was not limited. The document types were restricted to articles and review articles, and the languages were restricted to English. We used the following specific retrieval formula for advanced search: (TS=(HIV OR “human immunodeficiency virus”)) AND (TS=(“elite controller$” OR “elite suppressor$” OR “elite nonprogressor$” OR “HIV controller$”)). By combining manual screening with the “remove duplicates” function provided by CiteSpace 5.7.r5, we excluded unqualified documents according to the following exclusion criteria: (1) corrigendum documents; (2) duplicate publications; and (3) irrelevant documents.

### 2.2 Data export

All eligible documents were exported in plain text file format, and the record content was “full record and cited references.” The 1st to 500th documents were exported into a file named “download_1-500.txt,” and the 501st to 1000th documents were exported into another file named “download_501-1000.txt.”

### 2.3 Analysis

In the CiteSpace visualization figure, the node display pattern presents a tree ring structure, clearly representing the node’s history of occurrence or citation. The radius of each node is proportional to the total frequency of the node, and the thickness of each annual ring is proportional to the frequency of the node in that year. The thickness of each line indicates the cooperation, cocitation, or cooccurrence intensity between two nodes, and the color of each line indicates the year in which the cooperation, cocitation, or cooccurrence first occurred. As the color of the line changes from dark purple to light yellow, the first cooperation, cocitation, or cooccurrence time transitions from 2006 to 2022. Betweenness centrality quantifies the importance of a node in the network structure by measuring the proportion of the shortest pathways passing through a given node in a network (Chen, 2006). The higher the betweenness centrality of a node, the more essential it is in tying the whole network together and supporting the network framework. In particular, not all nodes with high frequency have high centrality, so it is necessary to distinguish between them. The node with high betweenness centrality (Centrality >= 0.1) has a purple ring, and the ring’s thickness describes the centrality value. In addition, the burst node decorated in red typically represents the transformation of a research field.

## 3 Results

### 3.1 Annual outputs of publications

**Figure 1:**
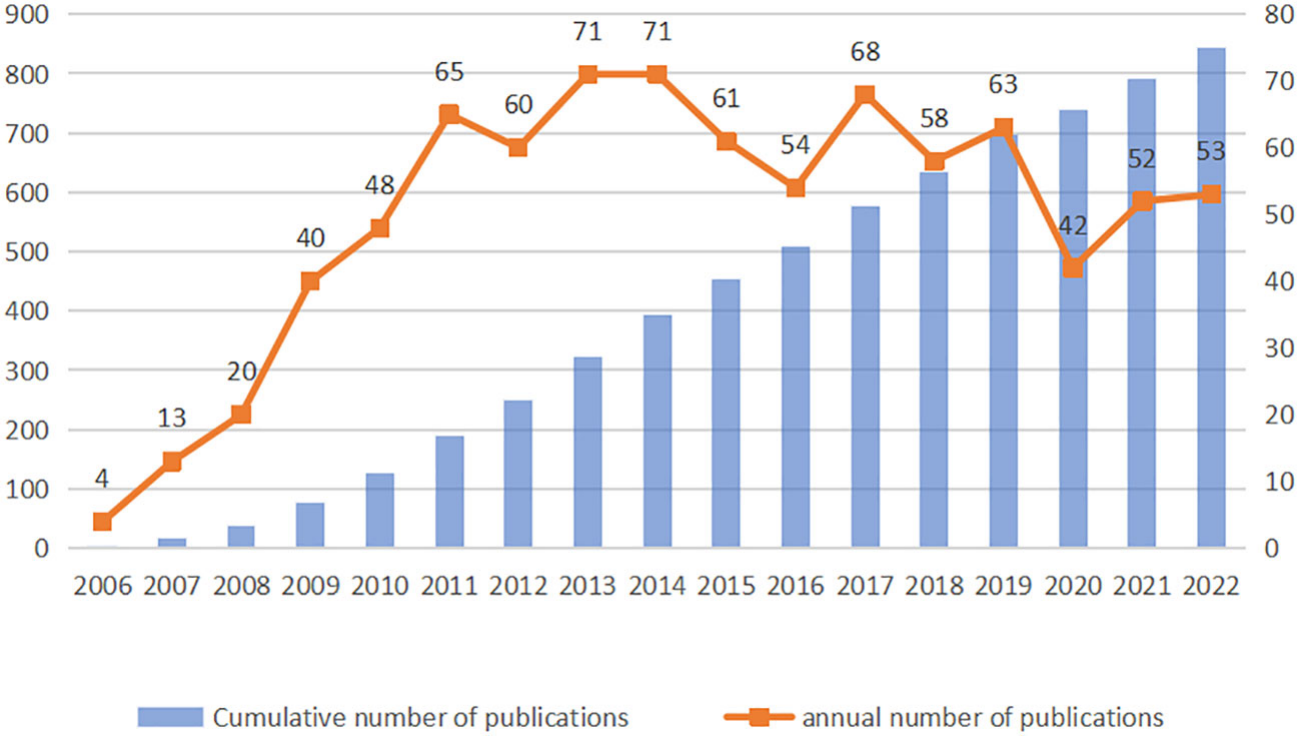
Trends in the annual and cumulative number of publications in the field of elite controllers.

The time variation of the annual outputs of publications on a study topic can provide insight into the research history of the field and forecast its future booms and busts. After excluding all ineligible papers, we finally included 843 relevant papers, the earliest of which was published by Bailey et al. in May 2006 (Bailey et al., 2006a). The change trends are shown in **Figure 1**.

Although this rare group of individuals was identified in the 1990s, it was not until October 1, 2005, that Lambotte et al. (Lambotte et al., 2005) first referred to this group as “HIV controllers.” However, this article was not included in our analysis because it was not included in WOSCC. Since then, the number of publications about elite controllers has increased substantially, from 4 in 2006 to 71 in 2014, indicating that elite controllers as a new concept has gradually attracted widespread attention. From 2014 to 2022, the number of published articles generally showed a declining tendency with some fluctuations, possibly due to the difficulty of sample discovery. Nevertheless, the number of articles published each year remained at a high level.

### 3.2 Distribution of countries/regions, institutions and authors

**Figure 2:**
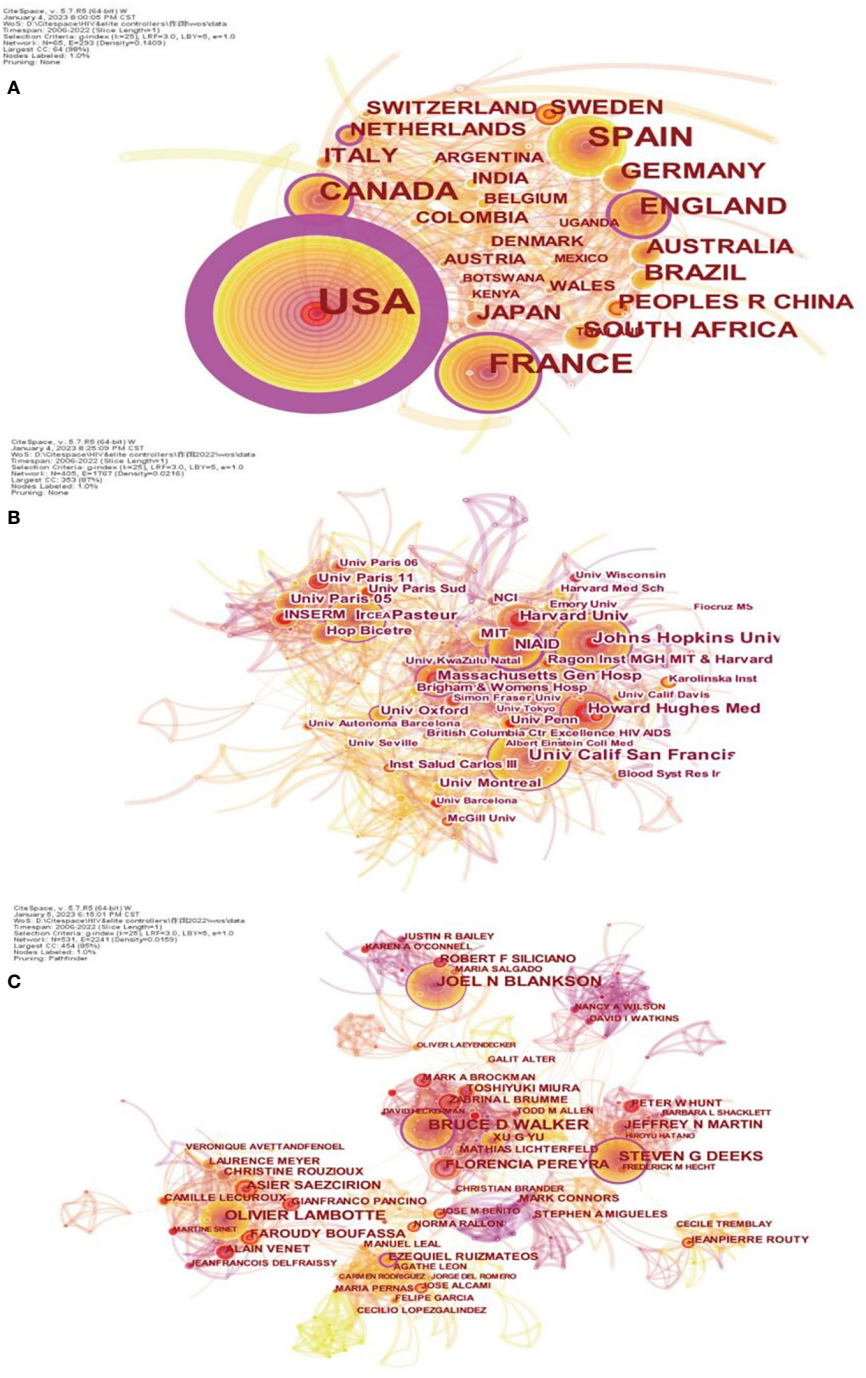
Citespace network map of publication distribution in the elite controller field. **(A)** The publication distribution and cooperation of 65 countries/regions. **(B)** The publication distribution and cooperation of 405 institutions. **(C)** The publication distribution and cooperation of 531 authors.

HIV is a serious contagious virus that can cause disease when left untreated thereby affecting global public health. Through joint research projects on elite controllers of various countries, institutions, and researchers, we can promote the extensive communication of academic information among multiple units, which will help find a breakthrough to combat HIV. CiteSpace was used to visualize the distribution and collaboration of countries, institutions, and authors. The 843 papers covered 65 countries, 405 institutions, and 531 authors. **Figures 2A-C** show the outcomes of cocountry, coinstitution, and coauthor relationships. **Figure 2A** contains 65 nodes and 293 lines with a density of 0.1409; **Figure 2B** contains 405 nodes and 1767 lines with a density of 0.0216; and **Figure 2C** contains 531 nodes and 2241 lines with a density of 0.0159. The size of a node represents the number of publications issued by a country, institution, or author. The larger the node, the greater the number of publications. The lines represent the cooperation between countries, institutions, or authors. The thicker the line, the greater the number of publications issued by the connected nodes in collaboration. The intricate links in the figures illustrate that close and complicated cooperative relations had been formed among different units in general.

**Table 1** shows the top 10 countries/regions ranked by publication quantity and betweenness centrality. The top countries/regions, based on quantity and centrality, were mainly located in Europe and North America. The USA (485), France (112), Spain (93), Canada (73), and England (60) occupied the top five in terms of quantity. The USA (1.10), England (0.19), France (0.12), Canada (0.11), and Netherlands (0.10) occupied the top five in terms of centrality. In particular, the United States accounted for 59% of the total published papers, and its betweenness centrality score also held a remarkable leading position.

**Table 1:** The top ten countries/regions with the most publications quantity (center sides) and betweenness centrality (right sides).

Rank	Country/region	Count (%)	Centrality	Country/region	Centrality	Count (%)
1	USA	485 (59.21%)	0.62	USA	1.10	485 (59.21%)
2	FRANCE	112 (13.67%)	0.06	ENGLAND	0.19	60 (7.33%)
3	SPAIN	93 (11.35%)	0.61	FRANCE	0.12	112 (13.67%)
4	CANADA	73 (8.91%)	0.06	CANADA	0.11	73 (8.91%)
5	ENGLAND	60 (7.33%)	0.67	NETHERLANDS	0.1	21 (2.56%)
6	BRAZIL	41 (5.01%)	0.06	BELGIUM	0.08	11 (13.4%)
7	GERMANY	38 (4.64%)	0.27	SPAIN	0.07	93 (11.35%)
8	SOUTH AFRICA	35 (4.27%)	0.06	BRAZIL	0.06	41 (5.01%)
9	AUSTRALIA	31 (3.79%)	1.06	GERMANY	0.04	38 (4.64%)
10	SWEDEN	28 (3.42%)	0.16	COLOMBIA	0.02	13 (1.59%)

The top 10 institutions are listed in **Table 2** according to publication quantity and betweenness centrality. Univ Calif San Francisco (87) contributed the most to publication quantity, while NIAID and Inst Pasteur (0.20) contributed the most to centrality. In the count ranking, the top 10 institutions came from monotonous sources, with 7 from the United States and 3 from France. However, the source of the top 10 institutions in the centrality ranking was more complex. In addition to 6 institutions from the USA that dominated the majority of the positions, there were 4 institutions from France, England, Spain, and Canada. These institutions with purple trims had high betweenness centrality and were crucial in intermediating and connecting the entire institutional cooperation, despite the relatively small number of publications of some institutions.

**Table 2:** The top ten institutions with the most publications (left sides) and betweenness centrality (right sides).

Rank	Institution	Count (%)	Centrality	Institution	Centrality	Count (%)
1	Univ Calif San Francisco	87 (10.32%)	0.16	NIAID	0.20	62 (7.35%)
2	Johns Hopkins Univ	84 (9.96%)	0.12	Inst Pasteur	0.20	58 (6.88%)
3	Howard Hughes Med Inst	62 (7.35%)	0.20	Massachusetts Gen Hosp	0.17	54 (6.41%)
4	NIAID	62 (7.35%)	0.10	Univ Calif San Francisco	0.16	87 (10.32%)
5	Inst Pasteur	58 (6.88%)	0.20	Univ Oxford	0.13	29 (3.44%)
6	Harvard Univ	55 (6.52%)	0.08	Johns Hopkins Univ	0.12	84 (9.96%)
7	Massachusetts Gen Hosp	54 (6.41%)	0.17	Howard Hughes Med Inst	0.10	62 (7.35%)
8	INSERM	38 (4.51%)	0.04	Univ Seville	0.09	14 (1.66%)
9	Univ Paris 05	35 (4.15%)	0.04	Harvard Univ	0.08	55 (6.52%)
10	MIT	34 (4.03%)	0.05	Univ Montreal	0.08	27 (3.20%)

**Figure 2C** shows the visual analysis of coauthors, and **Table 3** shows the top ten authors. Different from **Figures 2A, B**, the nodes in **Figure 2C** formed prominent teams of authors, in which the three largest teams, with Walker B.D., Blankson J.N., and Deeks S.G., as the core, had high publication volumes and high centrality at the same time. Their research results were of great reference value to scholars worldwide.

**Table 3:** The top ten authors with the most publications (left sides) and betweenness centrality (right sides).

Rank	Author	Count (%)	Centrality	Author	Centrality	Count (%)
1	Walker, Bruce D.	65 (7.71%)	0.31	Deeks, Steven G.	0.37	50 (5.93%)
2	Blankson, Joel N.	63 (7.47%)	0.13	Walker, Bruce D.	0.31	65 (7.71%)
3	Lambotte, Olivier	52 (6.17%)	0.02	Ruiz-Mateos, Ezequiel	0.25	22 (2.61%)
4	Deeks, Steven G.	50 (5.93%)	0.37	Blankson, Joel N.	0.13	63 (7.47%)
5	Pereyra, Florencia	36 (4.27%)	0.05	Prado, Julia G.	0.09	4 (0.47%)
6	Boufassa, Faroudy	32 (3.80%)	0.05	Meyer, Laurence	0.08	17 (2.01%)
7	Saez-Cirion, Asier	30 (3.56%)	0.00	Burwitz, Benjamin J.	0.08	5 (0.59%)
8	Martin, Jeffery N.	28 (3.32%)	0.05	Kaul, Rupert	0.07	2 (0.24%)
9	Siliciano, Robert F.	24 (2.85%)	0.00	Leal, Manuel	0.06	14 (1.66%)
10	Ruiz-Mateos, Ezequiel	22 (2.61%)	0.25	Allen, Todd M.	0.06	10 (1.17%)

### 3.3 Cocited authors and journals analysis

**Figure 3:**
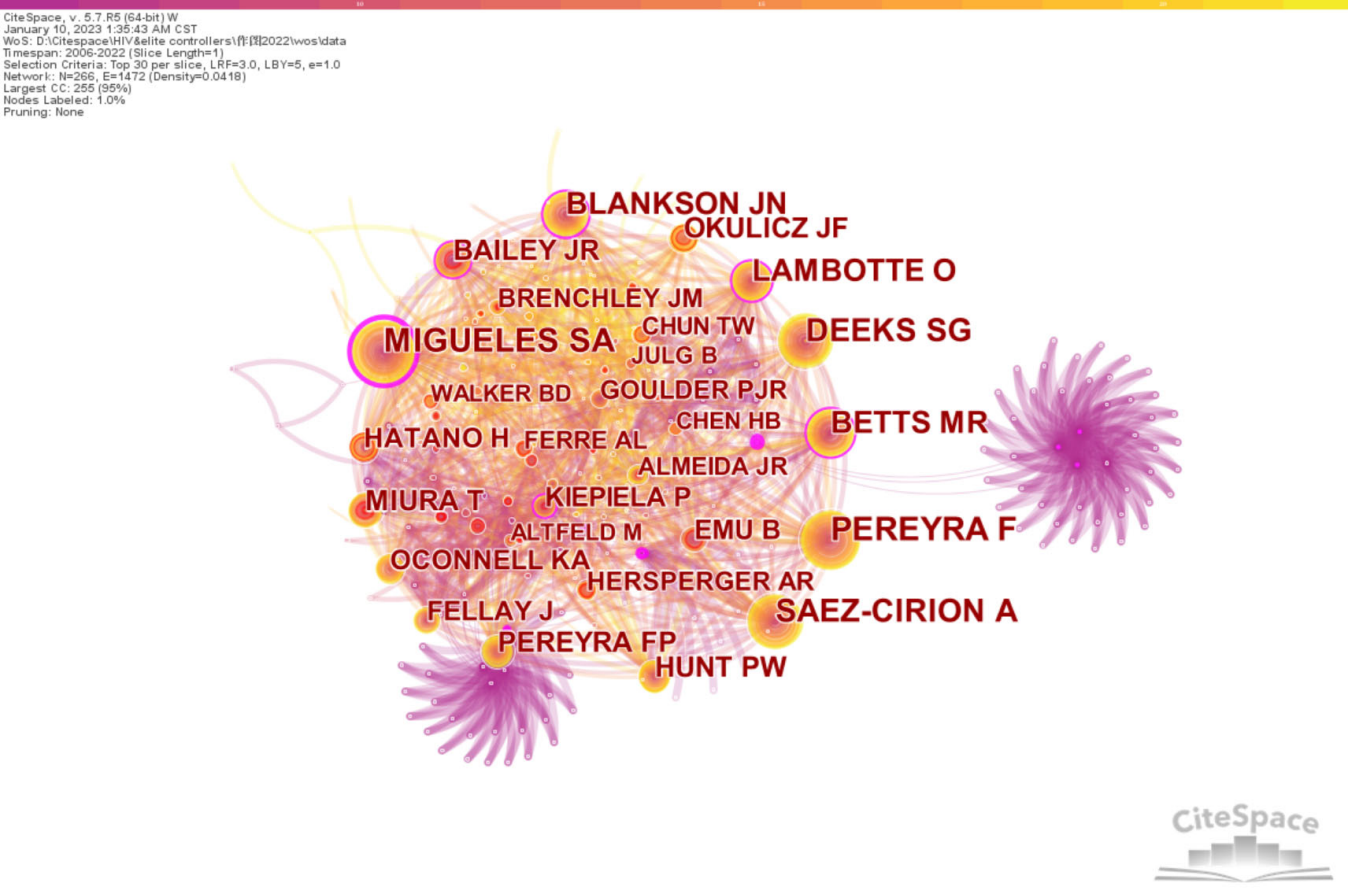
Visual analysis of 266 cocited authors in the field of elite controller research *via* CiteSpace.

**Figure 4:**
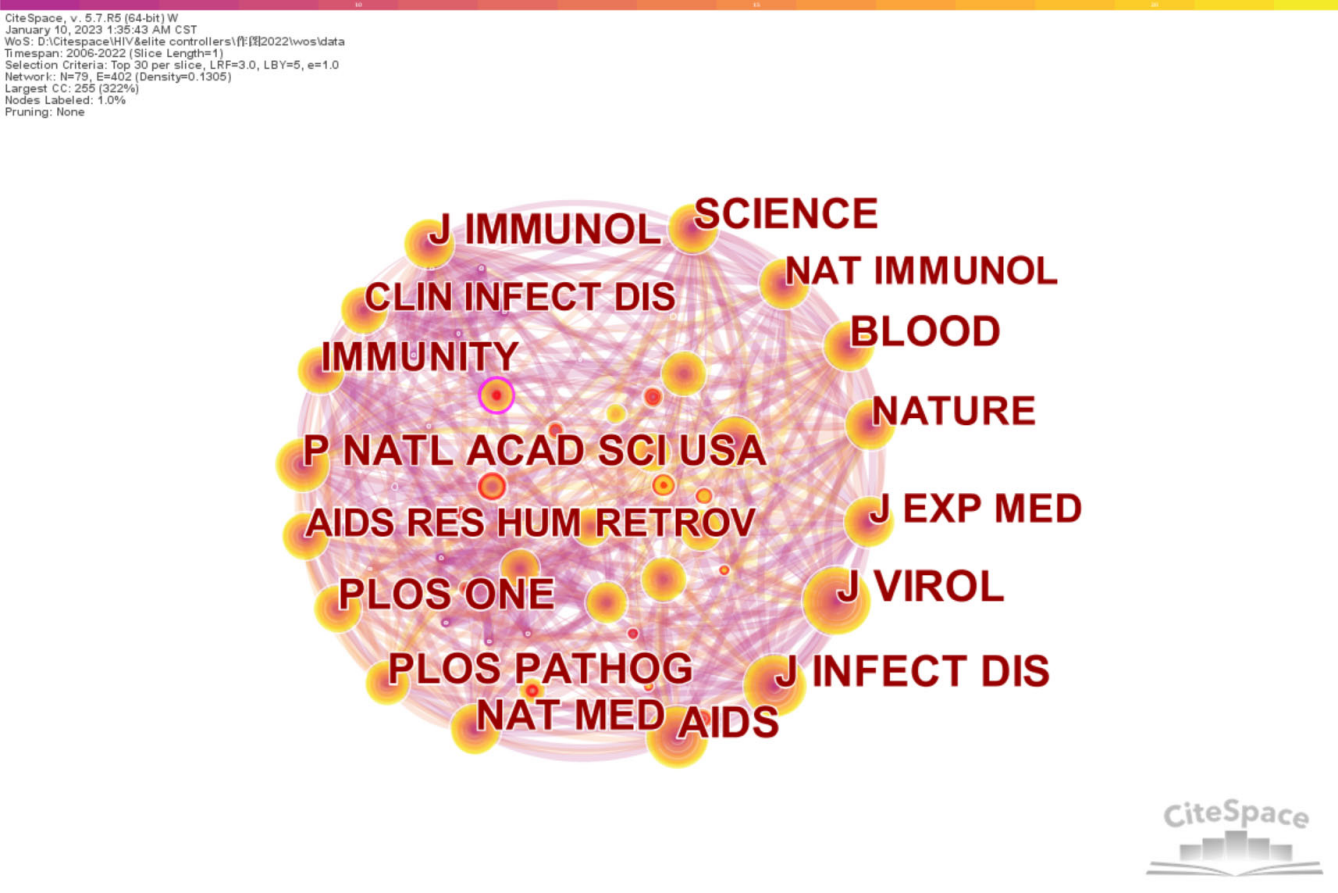
Visual analysis of 79 cocited journals in the field of elite controller research *via* CiteSpace.

Cocitation relationships refer to the phenomenon that two or more authors or journals are cited by one paper at the same time. The frequency of cocitations can be a reliable indicator to reflect the influence and authority of an author or journal in the knowledge domain. In total, publications on elite controller research included contributions from 266 cocited authors and 79 cocited journals. **Figure 3** exhibits the network of cocited authors, which contains 266 nodes and 1472 lines with a density of 0.0418. **Figure 4** exhibits the network of cocited journals, which contains 79 nodes and 402 lines with a density of 0.1305. The size of a node represents the total times of cocitations. The larger the node is, the more frequently the corresponding author or journal is cocited. Cocitation relationships exist between authors or journals connected by lines. The thicker the line is, the more cocitation counts between the connected authors or journals. Each node with a dramatic surge in cocitation at a particular period is decorated in red, and each node with strong betweenness centrality (centrality>=0.1) has a purple trim.

The top 10 authors and journals in terms of cocitations are listed in **Table 4**. Migueles S.A. (325) was the most cocited author, followed by Pereyra F. (320), Saez-Cirion A. (294), Deeks S.G. (290), and Betts M.R. (236). In addition, four journals had been cocited over 600 times: *Journal of Virology* (770), *Journal of Infectious Diseases* (675), *AIDS* (669), and *Proceedings of the National Academy of Sciences of the United States of America* (621). According to the 2021 Journal Citation Reports (JCR), the top ten cocited journals are all in regions Q1 and Q2.

**Table 4:** The top ten authors and journals with the most cocitations.

Rank	Cocited Author	Count	Cocited Journal	Count	IF (2021)	JCR
1	Migueles, Stephen A.	325	Journal of Virology	770	6.549	Q2
2	Pereyra, Florencia	320	Journal of Infectious Diseases	675	7.759	Q1
3	Saez-Cirion, Asier	294	AIDS	669	4.632	Q2
4	Deeks, Steven G.	290	Proceedings of the National Academy of Sciences of the United States of America	621	12.779	Q1
5	Betts, Michael R.	236	Science	564	63.714	Q1
6	Lambotte, Olivier	219	PLoS One	546	3.752	Q2
7	Blankson, Joel N.	197	Journal of Immunology	539	5.426	Q2
8	Hunt, Peter W.	180	Nature Medicine	536	87.241	Q1
9	Bailey, Justin R.	178	PLoS Pathogens	531	7.464	Q1
10	Miura, Tomoyuki	178	Blood	529	25.476	Q1

### 3.4 Reference cocitation

**Figure 5:**
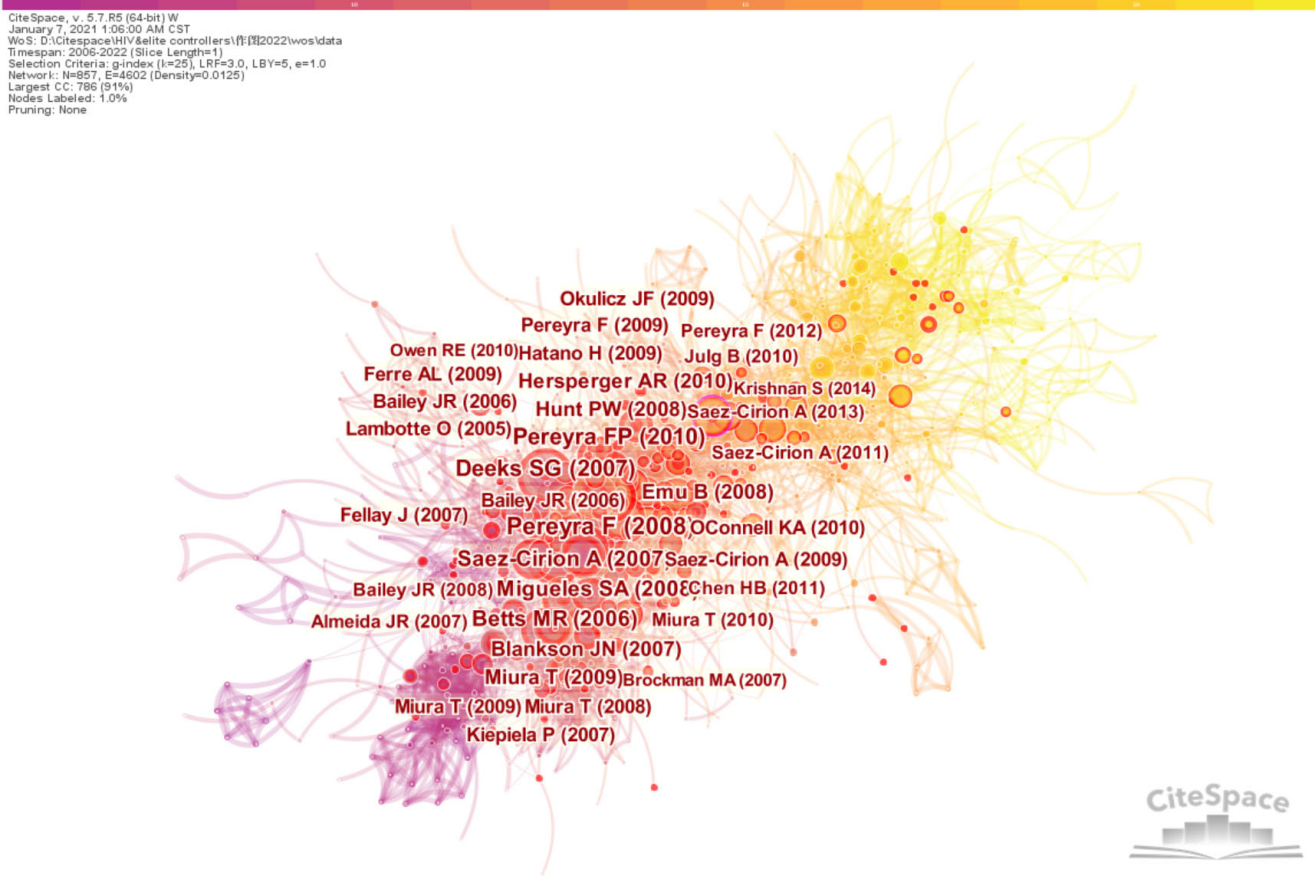
Visual analysis of 857 cocited references in the field of elite controller research *via* CiteSpace.

When an article cites two references simultaneously, there is a cocitation relationship between the two references. Compared with the frequency of citations, the frequency of cocitations can more accurately reflect the value of references, and references with high cocitation frequency often lay a solid foundation for the long-term exploration of the field. The 843 articles contained 857 cocited references altogether. **Figure 5** shows the map of cocited references in the elite controller area created by CiteSpace. The figure has 857 nodes and 4602 lines, and the network density is 0.0125. The size of each node represents the frequency of cocitation of a reference. The thickness of each line represents the intensity of the cocitation relationship between connected references. Different periods are visualized by different colored lines, meaning that the color of the line from dark purple to brilliant yellow represents the initial cocitation from early years to recent years. The red circles are used to emphasize the nodes with citation bursts, and the purple trims are used to emphasize the nodes with centrality greater than 0.1.

**Table 5** lists the top 15 references according to the frequency of cocitations, which unquestionably had enormous influence in the field of elite controllers. These references were all published between 2006 and 2010. Based on cocitation counts, Pereyra et al. (2008) ranked first with 118 cocitations, followed by Deeks and Walker (2007) with 94 cocitations, Pereyra et al. (2010) with 91 cocitations, Saez-Cirion et al. (2007) with 76 cocitations, and Migueles et al. (2008) with 75 cocitations.

**Table 5:** The top fifteen references with the most cocitations.

Rank	Count	Centrality	Cocited Reference	First Author (Year)
1	118	0.02	Genetic and immunologic heterogeneity among persons who control HIV infection in the absence of therapy	Pereyra et al. (2008)
2	94	0.01	Human immunodeficiency virus controllers: Mechanisms of durable virus control in the absence of antiretroviral therapy	Deeks and Walker (2007)
3	91	0.02	The Major Genetic Determinants of HIV-1 Control Affect HLA Class I Peptide Presentation	Pereyra et al. (2010)
4	76	0.03	HIV controllers exhibit potent CD8 T-cell capacity to suppress HIV infection ex vivo and peculiar cytotoxic T lymphocyte activation phenotype	Saez-Cirion et al. (2007)
5	75	0.04	Lytic Granule Loading of CD8+ T Cells Is Required for HIV-Infected Cell Elimination Associated with Immune Control	Migueles et al. (2008)
6	74	0.03	HIV nonprogressors preferentially maintain highly functional HIV-specific CD8^+^ T cells	Betts et al. (2006)
7	69	0.02	Isolation and characterization of replication-competent human immunodeficiency virus type 1 from a subset of elite suppressors	Blankson et al. (2007)
8	67	0.02	Relationship between T-cell activation and CD4^+^ T-cell count in HIV-seropositive individuals with undetectable plasma HIV RNA levels in the absence of therapy	Hunt, P.W. (2008)
9	65	0.01	HLA class I-restricted T-cell responses may contribute to the control of human immunodeficiency virus infection, but such responses are not always necessary for long-term virus control	Emu, B. (2008)
10	63	0.04	Perforin Expression Directly Ex Vivo by HIV-Specific CD8^+^ T Cells Is a Correlate of HIV Elite Control	Hersperger et al. (2010)
11	62	0.03	HLA-B57/B*5801 Human Immunodeficiency Virus Type 1 Elite Controllers Select for Rare Gag Variants Associated with Reduced Viral Replication Capacity and Strong Cytotoxic T-Lymphotye Recognition	Miura,T. (2009)
12	60	0.00	Maintenance of viral suppression in HIV-1-infected HLA-B*57^+^ elite suppressors despite CTL escape mutations	Bailey, J.R. (2006b)
13	58	0.03	Evidence for Persistent Low-Level Viremia in Individuals Who Control Human Immunodeficiency Virus in the Absence of Antiretroviral Therapy	Hatano et al. (2009)
14	54	0.04	A whole-genome association study of major determinants for host control of HIV-1	Fellay et al. (2007)
15	53	0.01	Persistent Low-Level Viremia in HIV-1 Elite Controllers and Relationship to Immunologic Parameters	Pereyra et al. (2009)

### 3.5 Reference bursts

**Figure 6:**
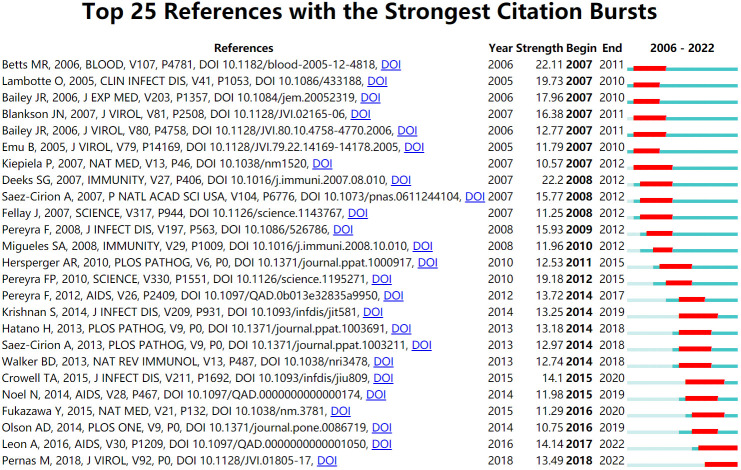
Top 25 references with the highest cocitation burst values of the 857 references on elite controller research. The red line corresponds to the period of surge in cocitation.

A reference has a high burst value when it is abruptly cocited heavily during a specific period, which often denotes that the research results of this reference represent innovative discoveries or new frontiers in this field. **Figure 6** shows the top 25 references with the strongest burst values and their relevant information. The dark blue line represents the start and end time when a reference is cocited, and the red line represents the period during which a reference continues to burst. The only two references with burst values over 20 came from Deeks et al. (Deeks and Walker, 2007) with a burst strength of 22.2 and Betts et al. (Betts et al., 2006) with a burst strength of 22.11. Deeks et al. also ranked 2 on the cocitation count list, and Betts et al. ranked 6. The subsequent three references had burst values of 19.73, 19.18, and 17.96. They came from Lambotte et al. (Lambotte et al., 2005), Pereyra et al. (Pereyra et al., 2010), and Bailey et al. (Bailey et al., 2006b), respectively. The latest burst reference was published in 2018 by Pernas et al. (Pernas et al., 2018), with a burst value of 13.48, which continues to burst.

By summarizing the major achievements of these strongest references, the research focus in the field of elite controllers can be reflected upon in four parts.

#### 3.5.1 Constructing a standardized definition of elite controllers

Lambotte et al. (Lambotte et al., 2005) described elite controller’s homogeneous characteristics and first named this unique phenotype “HIV controllers.” Blankson et al. (Blankson et al., 2007) successfully isolated and cultured replicable viruses with generally intact genes from some elite controllers, evidencing that some proportion of the elite controllers can control the replication of fully functional viruses over long periods rather than being infected with defective viruses. Olson et al. (Olson et al., 2014) proposed a more accurate identification criterion to classify elite controllers with less risk of disease progression, mainly referring to individuals with consistently undetectable HIV-RNA loads in plasma for ≥ 6 months or with > 90% of measurements < 400 copies/ml for ≥ 10 years.

#### 3.5.2 Understanding the mechanistic details of the elite controllers’ immune system

We still need to fully understand the decisive mechanism responsible for maintaining long term virological control. Although many common characteristics that may contribute to virologic control have been described among elite controllers, these characteristics are heterogeneous across individuals. No single factor has been an absolute predictor of HIV control thus far. Walker et al. (Walker and Yu, 2013) and Deeks et al. (Deeks and Walker, 2007), both high-impact reviews from top journals, systematically expounded the underlying mechanism of this rare phenotype from multiple perspectives and provided insights into its implications and inspiration. Bailey et al. (Bailey et al., 2006a) and Pereyra et al. (Pereyra et al., 2008) showed that elite controllers did not rely on high-titer neutralizing antibodies to suppress the virus. Pereyra et al. (Pereyra et al., 2008) and Saez-Cirion et al. (Saez-Cirion et al., 2007) demonstrated that elite controllers were distinguished by greater functionality rather than the breadth and magnitude of HIV-specific CD8^+^ T cell responses. Hersperger et al. (Hersperger et al., 2010) also highlighted the correlation between virologic control and the potent ability of HIV-specific CD8^+^ T cells to express perforin. Although Pereyra et al. (Pereyra et al., 2010) and Fellay et al. (Fellay et al., 2007) identified the significant contribution of human genetic variation, particularly HIA-B, to virologic control, nearly one-third of elite controllers lacked any of the protective HLA alleles known to delay disease progression (Pereyra et al., 2008). In addition, Bailey et al. (Bailey et al., 2006b), Migueles et al. (Migueles et al., 2008), and Krishnan et al. (Krishnan et al., 2014) also conducted in-depth discussions on the possible molecular mechanisms contributing to long term virological control.

#### 3.5.3 Developing and evaluating new treatments for HIV

Saez-Cirion et al. (Saez-Cirion et al., 2013) found that early and long-term cART enabled some individuals with risk-associated HLA alleles and worse CD8 T cell responses, compared to spontaneous controllers, to maintain prolonged viral control despite treatment interruption. Emu et al. (Emu et al., 2005) reported that the same immunological profile was shared between ART-treated controllers with drug-resistant HIV and untreated controllers. These results could provide insights into the search for a functional cure. Betts et al. (Betts et al., 2006) believed that maintaining highly functional HIV-specific CD8^+^ T cells is a critical indicator to evaluate the subsequent development of therapeutic drugs and vaccines, and the chemokine MIP-1 β may be the most accurate indicator to measure the frequency of responding CD8^+^ T cells. Kiepiela et al. (Kiepiela et al., 2007) observed that different viral proteins triggered different profiles of specific T cell responses, in which the breadth of Gag-specific responses was positively correlated with low viremia, whereas the breadth of Env-specific responses was inversely correlated with low viremia, providing references for vaccine exploration and evaluation. Fukazawa et al. (Fukazawa et al., 2015) studied elite controllers in rhesus monkeys infected with SIV and discovered that the exclusion of specific CD8^+^ T cells by B cell follicles could make B cell follicles sanctuaries for continuous viral replication, adding a hindrance to the development of vaccines and T cell immunotherapies.

#### 3.5.4 Discussing health risks faced by elite controllers

Despite the ability to control HIV replication without taking antiviral medication, elite controllers may still face risks to their health. In contrast, they have to respond to many challenges, mainly reflected in an increased probability of severe non-AIDS-related disease and loss of virological control. In a cohort study involving seven biomarkers, Noel et al. (Noel et al., 2014) discovered that elite controllers exhibited various inflammatory characteristics. In particular, interferon gamma-induced protein 10 (IP10), the most discriminant inflammatory biomarker, remained abnormally high and was positively and negatively associated with sustained T cell activation and CD4^+^ T cell count, respectively. Pereyra et al. (Pereyra et al., 2012) reported an association between elite controllers and a higher prevalence of atherosclerosis and expression of more immune activation markers. Crowell et al. (Crowell et al., 2015) also statistically found that elite controllers exhibited more frequent hospitalization, especially for cardiovascular disease, as compared to medically controlled patients. Hatano et al. (Hatano et al., 2013) considered that persistent low-level viral replication in elite controllers promoted immune activation and chronic inflammation while initiating ART can alleviate these phenomena. Leon et al. (Leon et al., 2016) proposed a baseline prediction score to identify elite controllers prone to experiencing disease progression. Pernas et al. (Pernas et al., 2018) discovered that a higher degree of viral diversity, decreased polyfunctionality of Gag-specific CD8^+^ T cells, and increased proinflammatory cytokine levels accurately predicted the virological loss of continuous control in transient elite controllers, a heterogeneous subgroup that accounted for approximately 28% of elite controllers. In particular, RANTES may be regarded as a new remarkable biomarker for the prediction of losing control virological control, and the immunoregulation of RANTES may be a therapeutic target for elite controllers.

### 3.6 Keyword timezone view and keyword bursts

Keywords are domain terms that briefly and concisely summarize the core theme and content of an article at a high level. Changes in hot keywords over time can roughly reflect changes in the research topics that receive the most attention. Timezone view analysis and burst detection of keywords in the field of elite controllers were conducted by CiteSpace. We analyzed the evolution and changes in research topics to predict future research hotspots and frontiers.

**Figure 7:**
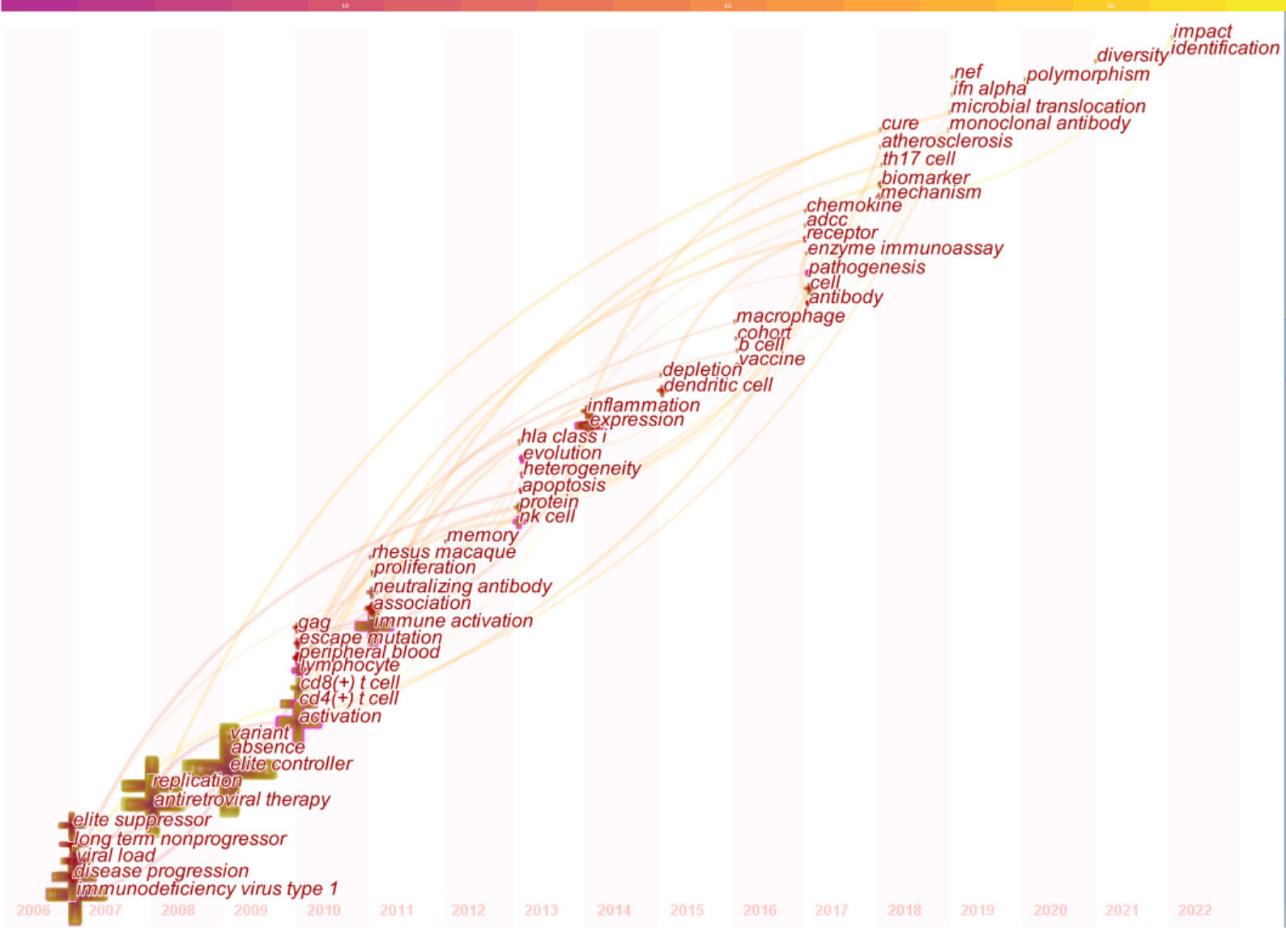
Timezone view based on cooccurrence status of the keywords in the elite controller field.

The keyword timezone view can clearly and intuitively illustrate the evolution process and stage characteristics of a knowledge domain in the time dimension by gathering keywords with the same initial appearance time in the same time zone. The size of the nodes represents the total frequency of keyword cooccurrence. The lines reflect the cooccurrence relationships between connected keywords. The thicker a line is, the more frequently the connected keywords appear in pairs in an article. **Figure 7** is the timezone view of the keywords performed by CiteSpace.

Based on the results of CiteSpace, we found that the focus of research on elite controllers varied at different stages. However, the study of their specialized immune and genetic mechanisms ran through the entire process, which was reflected by keywords such as “immune activation,” “hla class i,” “dendritic cell,” “expression,” “th17 cell,” and “polymorphism.” In 2011, “neutralizing antibody” initially appeared as a keyword. Since then, keywords such as “monoclonal antibody,” “cure,” and “impact” have gradually emerged, and there has been a growing number of articles describing elite controllers as inspiration for therapeutics that aim at a functional cure for HIV (Casado et al., 2020). It is worth mentioning that, albeit different from the extensive study of immune cells and gene expression, Koay et al. (Koay et al., 2018) found that spontaneous control of viruses was connected with a distinct and abundant gut microbiome. Vesterbacka et al. (Vesterbacka et al., 2017) mentioned the complex association between HIV infection, gut microbiota dysbiosis, and systemic inflammation and immune activation. They proposed that further study of the relationship between HIV and related microbiota and its metabolism could provide valuable strategies for achieving HIV remission and cure. Since 2014, while studying the mechanisms facilitating long term virological control, researchers have also paid attention to the price that elite controllers pay for long-term spontaneous virological control. “Inflammation,” “depletion,” and “atherosclerosis” became new high-frequency keywords. Sanchez et al. (Sanchez et al., 2015) found that CD4^+^ T cell depletion due to lymphoid tissue fibrosis was present in both controllers and noncontrollers. Cockerham et al. (Cockerham and Hatano, 2015) also argued that persistent inflammation, CD4^+^ T cell depletion, and possibly inflammation-related cardiovascular disease are unwanted effects that can coincide with long term virological control and thus possible coincide a functional cure. Sepulveda-Crespo et al. (Sepulveda-Crespo et al., 2022) proved that high sTNF-R1 was a plasma biomarker for identifying elite controllers who lost the innate control of HIV replication.

**Figure 8:**
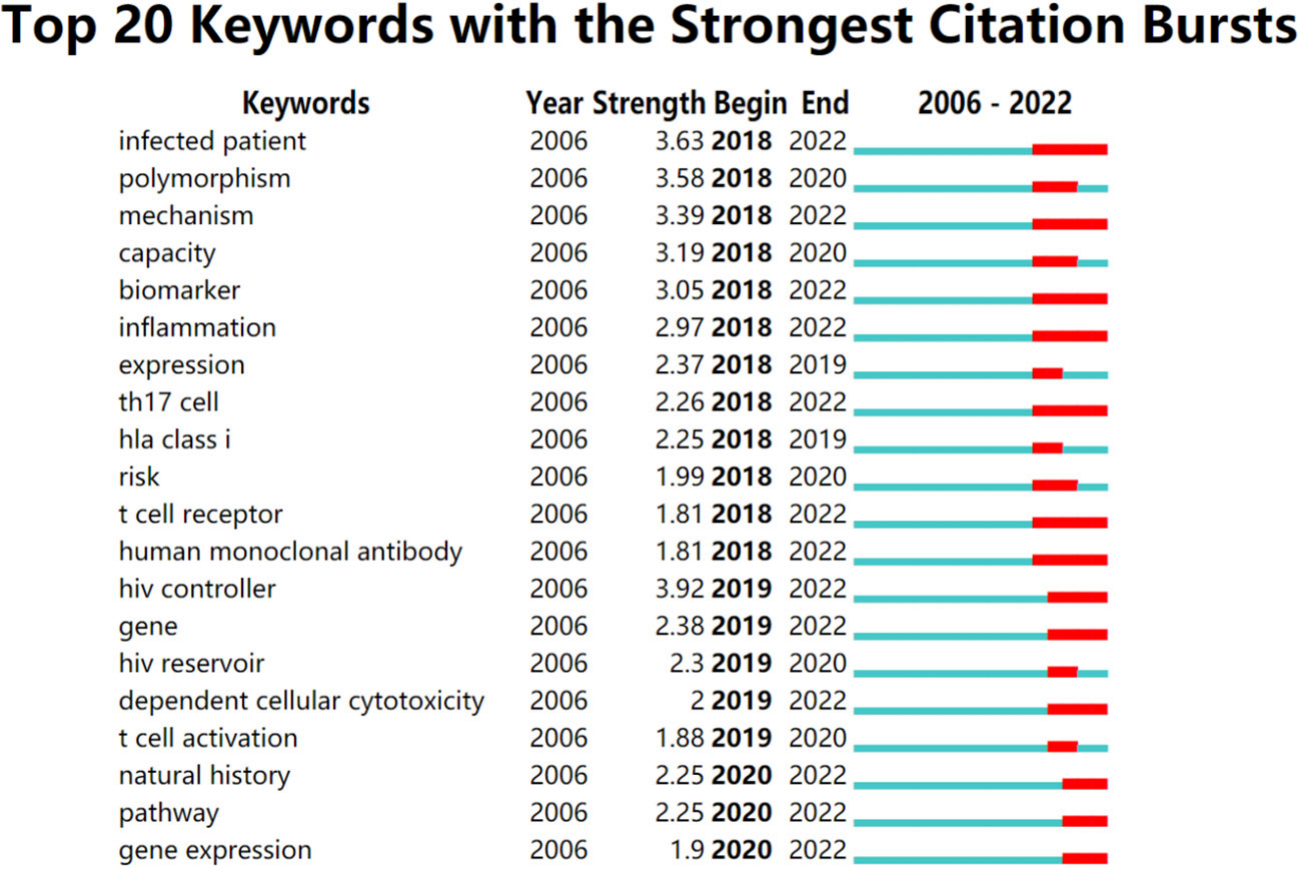
Top 20 keywords with the strongest burst values in the last 5 years on elite controller research. The red line corresponds to the period of surge in cooccurrence.

With strong surges in cooccurrence, burst keywords reflect the abrupt turning points of the concerned research directions to some extent. The frontiers of research are advancing chronologically. Early burst keywords can reflect early research frontiers, and current burst keywords can predict future research hotspots. Here, we listed the top 20 keywords with the strongest burst values over the past 5 years in **Figure 8**, among which “infected patient,” “mechanism,” “biomarker,” “inflammation,” “th17 cell,” “t cell receptor,” “human monoclonal antibody,” “HIV controller,” “gene,” “dependent cellular cytotoxicity,” “natural history,” “pathway,” and “gene expression” are still experiencing ongoing bursts at present. These keywords may indicate the research hotspots of the future.

### 3.7 Keyword clusters

**Figure 9:**
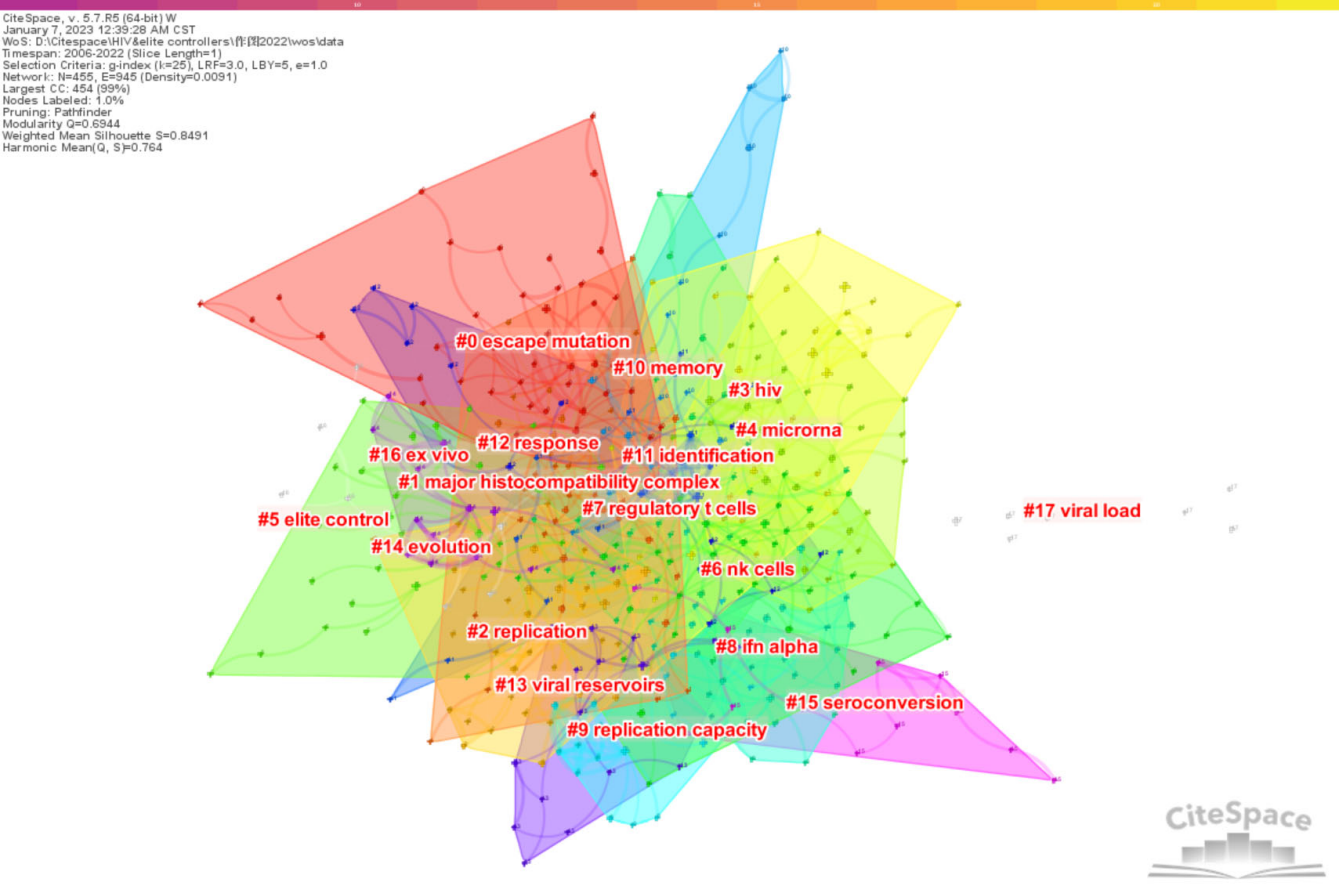
Clustered networks based on cooccurrence status of the keywords *via* CiteSpace. Red words are cluster labels extracted by LLR algorithm.

To represent the research frontiers corresponding to certain knowledge bases, the spectral clustering algorithm was used to cluster keywords, and the log-likelihood rate (LLR) algorithm was used to extract the keywords from citing articles to label clusters. CiteSpace adopts the modularity (*Q*) and the mean silhouette (*S*) as indexes to evaluate the clustering quality. Both of them are valued within the interval [0, 1). Generally, when *Q* values are over 0.3, clusters develop a significant community structure, namely, dense connections within clusters and sparse connections among clusters. When *S* values are greater than 0.7, the nodes within the clusters have an outstanding degree of homogeneity, and the clustering reliability is high. In **Figure 9**, CiteSpace generated 18 clusters with 455 nodes and 945 connections. From cluster #0 to #17, the clusters shrank successively. The largest cluster was cluster #0, containing 44 nodes and accounting for 9.67% of the whole network. The smallest cluster was cluster #17, containing 8 nodes and accounting for 1.76% of the whole network. The *Q* value of the network was 0.6944, indicating that the clusters were divided into a satisfactory structure. The mean *S* of all clusters was 0.8491, denoting that the results had a convincingly high efficiency. Currently, active clusters include cluster 1 (“major histocompatibility complex”), cluster 2 (“replication”), cluster 6 (“nk cells”), and cluster 8 (“ifn alpha”). The specific information of the clusters is shown in **Table 6**. The keywords attached to these four clusters may indicate the direction of future research.

**Table 6:** Detailed information on active keyword clusters in the elite controller field.

Cluster ID	Size	Sillouette	Mean year	Activeness	Major keywords
1	42	0.618	2013	Active	major histocompatibility complex; gp120; loss of control; pathogenesis; chemokine receptor; binding; samhd1; neutralization; microbiome; immune reconstitution;
2	37	0.956	2009	Active	replication; *in vivo*; CCR5 density; determinant; class i molecule; antibody profiling; HAART interruption; discontinuation; glucose; acute phase;
6	32	0.847	2013	Active	NK cells; homeostasis; AIDS vaccine; innate immunity; B cells; mucosal; CTL escape; broad; plasmacytoid dendritic cell; T-cell depletion;
8	30	0.871	2016	Active	IFN alpha; PD 1 expression; ADCC; efficacy; CD39; inflammation; feature; expansion; HIV reservoir; antibody dependent function;

## 4 Conclusion and perspectives

In this study, CiteSpace software was used to conduct a bibliometric analysis of people living with HIV and maintaining long term virological control without taking antiviral medication, also referred to as elite controllers, publications included in WOSCC up to 2022. We systematically illustrated the general landscapes, core research hotspots, and frontier development trend in this field from eight aspects: publication outputs, countries/institutions/authors cooperation networks, authors/journals cocitation networks, reference cocitation, reference bursts, keyword timezone view, keyword bursts, and keyword clusters.

Since the elite controllers were initially named in 2005, this research field has garnered extensive interest from scholars and produced remarkable achievements. Here, we identified the most productive countries, institutions, and authors. They have established representative research teams with close internal cooperation, providing solid academic support for subsequent research. We also identified the top ten influential authors and journals with the most cocitations, whose published articles are highly recognized by the academic community.

Based on the analysis of references and keywords, the research hotspots and frontiers in the elite controller field are described in the following three parts.

### 4.1 Deciphering the mechanisms of durable control

Our study indicates that the research hotspots for durable control of HIV in elite controllers can be specifically reflected by “HLA,” “dendritic cell,” “NK cells,” “th17 cell,” and other often cocited keywords. Current studies have not fully unraveled the decisive mechanisms of elite controller formation. However, many studies have revealed that restricting HLA class I alleles and strong CTL responses are the dominant components of forming processes. Additional triggers are also involved in modulating the effective control of virus replication. Limited studies have shown that the distinguished effector function of NK cells can be a hallmark of this phenotype (Vargas-Inchaustegui et al., 2012; Marras et al., 2013). In addition, the strong immune recognition of HIV-1 by dendritic cells can effectively stimulate the multifunctional T cell responses, which may be the reason for the potent T cell immune responses in elite controllers (Martin-Gayo et al., 2015). Recent ground-breaking researches also point to elite controllers’ specificity of integration sites of latent viral reservoirs. Lian et al. (Lian et al., 2021) revealed that defective proviruses in elite controllers frequently positioned in euchromatin locations where DNA transcription was active, whereas intact proviruses commonly integrated into heterochromatin regions to resist immune targeting and elimination and confer long-lasting deep latency. Furthermore, an extraordinary antiviral immune activity could effectively eliminate many proviral species and was associated with a reduced frequency of proviral mutations within elite controllers. Jiang et al. (Jiang et al., 2020) also found that the proviral sequences of elite controllers were commonly located in or surrounded by non-coding regions composed of dense heterochromatin, termed “gene desert.” Nevertheless, it is important to stress that none of the aforementioned factors provide complete and conclusive explanations for virus control. Future exploration needs to dissect the underlying mechanisms of the spontaneous and consistent control of HIV replication in elite controllers.

### 4.2 Delineating the implications for the development of treatments for HIV infection

Elite controllers demonstrate that sustained virological control without antiviral treatment is possible, and thus a functional cure for other people living with HIV may therefore be possible, which can be specifically reflected by “antibody,” “cure,” “vaccine,” and other often cocited keywords. They provide ideas for novel therapeutics and prophylactic vaccine approaches. Although there is significant heterogeneity within elite controllers, we can explore the possibility of a solution for the majority of PLHIV to achieve a functional cure by investigating the typical immune characteristics among the elite controllers and non-controllers and conducting genome-wide identifications of genetic susceptibility to the virus. In addition, researchers are relying on elite controllers to screen potent broad-spectrum neutralizing antibodies, providing bright prospects for HIV vaccine development. Kong et al. (Kong et al., 2016) isolated broadly neutralizing antibodies, termed VRC01-Class antibodies, from a Chinese elite controller that can target the conserved and vulnerable CD4-binding site to resist diverse HIV-1 strains.

### 4.3 Highlighting the clinical risks faced by elite controllers

Unfortunately, elite controllers are associated with several clinical risks, including “inflammation,” T cell “depletion,” “atherosclerosis,” “loss of control,” and others. The suppression of the virus by elite controllers is not complete or permanent. Low-level viremia can still be detected in most elite controllers by ultrasensitive assays (Pereyra et al., 2009 and Hatano et al., 2009). With the possibility of virus evolution facilitating immune escape, combined with the possibility of declined CD4+ T cell counts, elite controllers are facing the challenge of losing control of virus replication. Persistent inflammation and immune activation may also lead to a higher prevalence and mortality of non-AIDS-defined diseases in elite controllers than in post-treatment controllers (PTCs). Studies have demonstrated significant improvements in laboratory indexes when antiviral treatment is started in elite controllers. Therefore, in the future, it is urgent to design authoritative guidelines for elite controllers to consider whether to initiate ART or other therapies based on viral load, immune function, comorbidity, and other criteria to improve their clinical outcomes.

In conclusion, this study is helpful for scholars to quickly and intuitively understand the present status and trends and provides different perspectives to identify domain problems. However, this study also has some limitations. Prior to 2005, researchers had identified the existence of elite controllers but had not named them separately from the blanket term long-term nonprogressors (LTNPs) (Migueles et al., 2000; Migueles et al., 2002). As a result, we missed some of the most important and most cited early articles. Some of the included articles not only studied elite controllers but also involved the descriptions of other PLHIV, LTNPs, and PTCs, which may affect the accuracy of the research results. In addition, there are certain limitations to the CiteSpace software. For instance, it cannot recognize the authorship classification, so all authors of an article will be identified equally. Nevertheless, this study will assist us in locating landmark and groundbreaking articles, recognizing the mainstream focus and frontier hot spots in this domain, and serving as a reference for subsequent relevant research.

## Data availability statement

The original contributions presented in the study are included in the article/supplementary material. Further inquiries can be directed to the corresponding author.

## Author contributions

XY and YL conceived and designed the study. XY collected and analyzed the data and wrote the original manuscript. YL revised and polished the manuscript. All authors contributed to the article and approved the submitted version.
